# Thermodynamic Scaling of the Dynamics of a Strongly Hydrogen-Bonded Glass-Former

**DOI:** 10.1038/s41598-017-01464-2

**Published:** 2017-05-02

**Authors:** Michela Romanini, María Barrio, Roberto Macovez, María D. Ruiz-Martin, Simone Capaccioli, Josep Ll. Tamarit

**Affiliations:** 1 Grup de Caracterització de Materials, Universitat Politècnica de Catalunya, EEBE, Departament de Física, and Barcelona Research Center in Multiscale Science and Engineering, C. Eduard Maristany 10-14, E-08019 Barcelona, Spain; 2 Dipartimento di Fisica, Università di Pisa, and IPCF-CNR, Largo B. Pontecorvo 3, I-56127 Pisa, Italy

## Abstract

We probe the temperature- and pressure-dependent specific volume (*v*) and dipolar dynamics of the amorphous phase (in both the supercooled liquid and glass states) of the ternidazole drug (TDZ). Three molecular dynamic processes are identified by means of dielectric spectroscopy, namely the α relaxation, which vitrifies at the glass transition, a Johari-Goldstein ***β***_JG_ relaxation, and an intramolecular process associated with the relaxation motion of the propanol chain of the TDZ molecule. The lineshapes of dielectric spectra characterized by the same relaxation time (isochronal spectra) are virtually identical, within the studied temperature and pressure ranges, so that the time-temperature-pressure superposition principle holds for TDZ. The α and ***β***_JG_ relaxation times fulfil the density-dependent thermodynamic scaling: master curves result when they are plotted against the thermodynamic quantity *Tv* ^*γ*^, with thermodynamic exponent γ approximately equal to 2. These results show that the dynamics of TDZ, a system characterized by strong hydrogen bonding, is characterized by an isomorphism similar to that of van-der-Waals systems. The low value of γ can be rationalized in terms of the relatively weak density-dependence of the dynamics of hydrogen-bonded systems.

## Introduction

It has been found in recent years that van-der-Waals and other simple material systems are characterized by the existence of a simple scaling of structural and molecular dynamic properties with the density, in the sense that isomorphic curves can be found along which the structural parameters and dynamics are invariant, *i*.*e*., they can be rescaled on a single master curve when plotted against suitable density-dependent reduced units^1–3^. For glass-forming materials, such approach, called “hidden scale-invariance” or “thermodynamic scaling”, originates from the observation that a combined description can be given of the pressure and temperature dependence of the dynamics of van-der-Waals and other simple glass formers, known as “Roskilde-simple liquids”. These are characterized, as a result of specific features of the intermolecular interactions^3^, by near proportionality between the isochoric thermal equilibrium fluctuations of the virial and the potential energy. In particular, the relaxation time (*τ*) of certain dynamic processes can be plotted independently of the thermodynamics conditions onto a single master curve^3–10^, described by:

1 $$\mathrm{Log}(\tau )=f(T{v}^{\gamma }).$$

Here *v* is the specific volume (inverse density), *γ* is a material constant, and the function *f* might be an Arrhenius equation or a modification thereof.

Such simple and appealing idea for the universal description of molecular dynamics in glass formers is, however, considered to fail^11–13^ in the case of strongly hydrogen-bonded systems such as water^12^ or dipropylene glycol^14^ and strongly associating liquids and polymers in general. This behavior is believed to be a consequence of the geometrical constraints imposed by hydrogen-bonding or covalent interactions, which have specific directions: the hidden scale invariance is apparently incompatible with directional interactions, so hydrogen-bonded and covalently bonded systems are not expected to be Roskilde-simple liquids^3^. In the case of water, for example, the directional intermolecular interactions in the hydrogen bond network give rise to an open structure of lower density than unassociated water molecules, and application of pressure is believed to disrupt the network equilibrium^15^.

In this contribution we characterize experimentally the molecular dynamics of the strongly hydrogen-bonded ternidazole drug (3-(2-methyl-5-nitroimidazol-1-yl)-propan-1-ol, C_7_H_11_N_3_O_3_, hereinafter TDZ)^16, 17^ in its amorphous state (supercooled liquid and glass). Hydrogen bonding in TDZ is so strong that the crystalline phase of TDZ consists of tetramers held together by intermolecular hydrogen bonds involving the oxygen moieties^18^. We find that both the cooperative molecular relaxation associated with the glass transition (α), and the related precursor Johari-Goldstein relaxation (*β*_JG_), obey power-law density scaling when the relaxation times are plotted against the thermodynamic quantity *Tv* ^*γ*^ with γ ≈ 2. A master-curve scaling is observed also when the characteristic times are displayed against the normalized temperature *T*/*T*_g_. The thermodynamic system-specific exponent γ is the same for both α and *β*_JG_ dynamic processes, and it is independent of the particular (*P*,*T*) conditions of the measurements. These results show that TDZ exhibits “isomorphs” as those reported in amorphous and crystalline van-der-Waals systems^3, 19–22^. The existence of such isomorphs for the dynamics of TDZ is *a priori* unexpected for a system with strong hydrogen bonds. The obtained value of the thermodynamic exponent *γ* ≈ 2 is quite low, which is in agreement with the fact that variations of density (specific volume) have less impact on hydrogen-bonded systems than simple liquids, due to the competing effect of a reduced intermolecular spacing and the bending of hydrogen bonds^23–25^. The value of *γ* for TDZ is in fact the lowest ever reported for a molecular glass-former obeying, over a wide *T*-*P* interval, thermodynamic scaling, isochronal loss peak invariance, and co-invariance of α and *β*_JG_ relaxation times.

**Figure 1:**
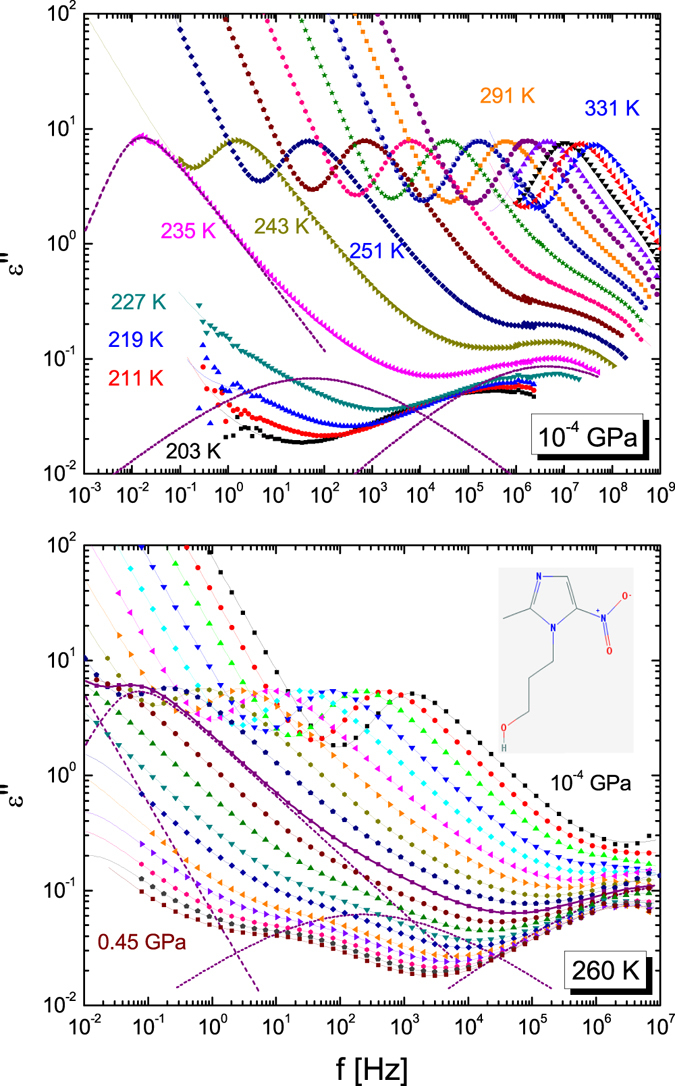
Dielectric loss spectra of supercooled TDZ for several temperatures at normal pressure (upper panel) and for pressures between normal pressure and 0.45 GPa every 25 MPa at the constant temperature of 260 K (lower panel). In both panels, markers are experimental points, continuous lines are fits, and dashed lines represent separate spectral components (*i*.*e*., the conductivity term and a separate peak for each relaxation). For clarity, the fit components are only shown for the spectrum acquired at 235 K in the upper panel, and for the spectrum at 0.225 GPa in the lower panel. Inset to the lower panel: molecular structure of the TDZ molecule.

Figure 1 displays several examples of dielectric loss spectra acquired on supercooled TDZ for a set of selected temperatures at ambient pressure (upper panel) and for a set of selected pressures at the constant temperature of 260 K (lower panel). The molecular structure is shown as inset to the lower panel. The logarithmic spectra display a linear background at low frequency, corresponding to the dc conductivity signal, followed by a prominent loss associated with the collective diffusional motions of the TDZ molecules (α relaxation). At yet higher frequencies two less intense secondary relaxations are observed (*β* and γ processes).

The dielectric loss spectra were fitted as the sum of three components: for the primary relaxation, a Cole-Davidson function was employed, defined as ${\varepsilon }_{CD}(f)={\varepsilon }_{\infty }+{\rm{\Delta }}{\rm{\varepsilon }}/{(1+i2\pi f{\tau }_{\alpha })}^{d}$, where τ_α_ is the characteristic time of the α relaxation^26^; for each secondary process, a Cole-Cole function was used, given by ${\varepsilon }_{CC}(f)={\varepsilon }_{\infty }+{\rm{\Delta }}{\rm{\varepsilon }}/[1+{(i2\pi f\tau )}^{c}]$ ^27, 28^. The exponents *c* and *d* determine the width of the corresponding loss feature. Additionally, a term ${\sigma }_{dc}/(i{{\rm{\varepsilon }}}_{0}2\pi f)$, taking into account the d.c. conductivity contribution, was added to the imaginary part of permittivity.

**Figure 2:**
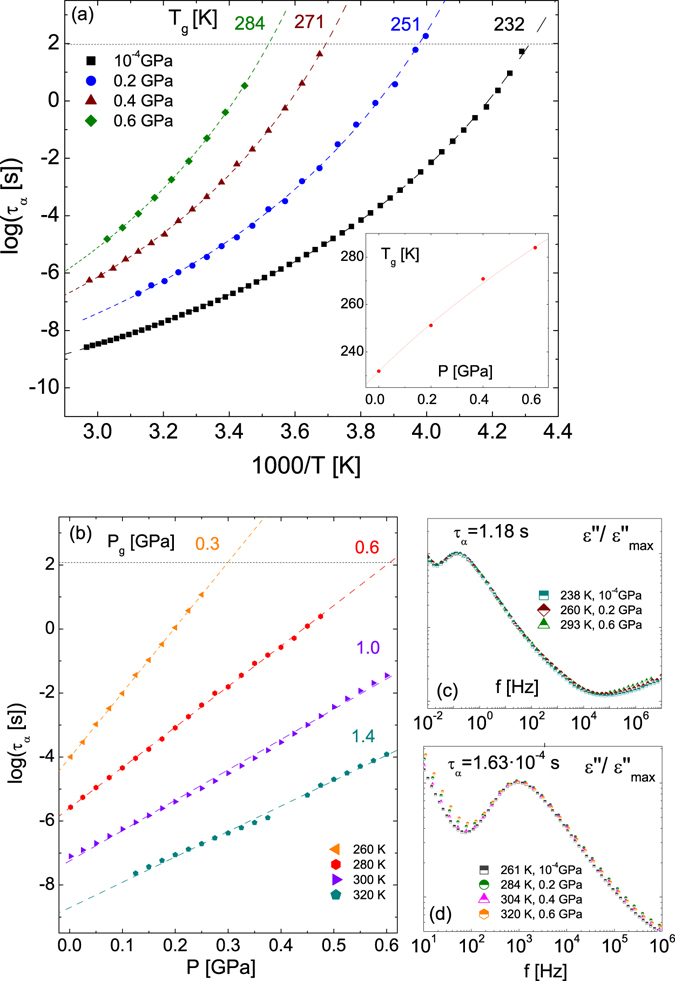
(**a**,**b**) Relaxation times of the α process as a function of the reciprocal temperature for a set of four pressures (**a**), and as a function of pressure, for a set of four temperatures (**b**), as indicated . The dotted lines correspond to fits with the Vogel-Furcher-Tammann Eq. 2 in panel (a) and with the pressure-dependent Arrhenius Eq. 4 in panel (b). Inset to (**a**): dependence of the glass transition temperature upon pressure for the data in (**a**), and fit according to Eq. 3. (**c**,**d**) Series of dielectric loss spectra for two sets of different pressure and temperature conditions, corresponding to the same α-relaxation time τ_α_ = 1.18 s (**c**) and τ_α_ = 1.63 · 10^−4^ s (**d**). The data were normalized to the same dielectric strength and shifted horizontally only slightly to allow a better visual comparison of the spectra.

We first focus on the primary (α) process. The relaxation times τ_α_ of the primary relaxation are plotted in Fig. 2 as a function of temperature for different pressures (Fig. 2(a)) and as a function of pressure for different temperatures (Fig. 2(b)). The temperature dependence of the α relaxation time τ_α_ is more pronounced than a (simply activated) Arrhenius behaviour, and it was modelled with the Vogel-Fulcher-Tammann (VFT) equation, given by^29^:

2 $${\tau }_{\alpha }(T)={\tau }_{\infty }\exp [s\,{T}_{{\rm{VF}}}/(T-{T}_{{\rm{VF}}})]$$

Here the prefactor *τ*_∞_, the strength parameter *s* and the so-called Vogel-Fulcher temperature *T*_VF_ are phenomenological constants. From the VFT fits of the isobaric relaxation times with Eq. 2, it is possible to calculate the glass temperature *T*_g_ for every isobar, as indicated in Fig. 2(a). As expected, the glass transition temperature is higher at higher applied pressure. For the majority of glass-formers, the variation of T_g_ with pressure is sublinear and can be described by means of the Andersson and Andersson empirical equation, given by^30^:

3 $${T}_{g}={k}_{1}{(1+{k}_{2}P)}^{{k}_{3}}$$

where k_1_, k_2_ and k_3_ are material constants. The inset to Fig. 2(a) shows that this equation holds for the case of TDZ. The pressure-dependence of the relaxation time τ_α_ appears instead to display a simply activated behaviour. The data of Fig. 2(b) were fitted using the pressure-dependent Arrhenius equation:

4 $${\tau }_{\alpha }(P)={\tau }_{0}\exp (P{V}_{{\rm{a}}}/RT)$$

Here *R* is the gas constant, *τ*_0_ is the value of *τ*_α_ at atmospheric pressure and *V*_a_ is the molar activation volume. A simple glance at Fig. 2(b) reveals that the slope of the Log(*τ*_α_) curves, and therefore the activation volume, is lower at higher temperature. This is expected since the free volume necessary for a molecular reorientation to occur is lower the higher the temperature. In an analogous fashion as for the isobaric measurements, the fits of the pressure-dependent (isothermal) data with Eq. 4 allow one to calculate, for a given temperature, the glass transition pressure *P*_g_, which is defined as the pressure at which the system displays a relaxation time of 100 seconds. As it may be gathered from Fig. 2(b), *P*_g_ increases with increasing temperature as expected.

The most relevant parameters of the primary relaxation associated with the glass transition, as extracted from the fits, are listed in Table 1. The last column in this table shows the values of the exponent *β*_KWW_ of the stretched exponential function that describes the α spectral feature in the time domain. The stretched exponent was estimated, using the Alvarez-Alegra-Colmenero relation^31, 32^, as ${\beta }_{{\rm{KWW}}}={d}^{1/1.23}$, where *d* is the exponent of the Cole-Davidson function used to fit the α relaxation. The relatively small value of *β*_KWW_ indicates a wide distribution of relaxation times, which in turns points to a strong cooperative character of the α process (the more cooperative the α process, the smaller the value of the stretched exponent^33^. It is worth pointing out that *β*_KWW_ has a decreasing trend on approaching the glass transition, both over isobaric cooling and isothermal compressions, revealing an increasingly cooperative character of the relaxation process or an increasing dynamic heterogeneity.

**Table 1:** Values of the glass transition temperature (*T*_g_) and pressure (*P*_g_), fragility (*m*), activation volume (*V*_a_) and range of *β*_KWW_ parameter both for isobaric (top) and isothermal (bottom) measurements.

*P* [GPa]	*T*_g_ [K]	*m*	*β*_KWW_
10^−4^	231.9 ± 0.1	76.8 ± 1.6	0.60–0.96
0.2	251.2 ± 0.2	73.3 ± 1.1	0.60–0.74
0.4	270.8 ± 0.3	71.0 ± 1.4	0.60–0.73
0.6	284.0 ± 0.5	66.1 ± 1.1	0.60–0.68
***T*** **[K]**	***P***_**g**_ **[MPa]**	***V***_**a**_	***β***_**KWW**_
260	296 ± 1	101.5 ± 0.4	0.60–0.67
280	600 ± 5	67.9 ± 0.4	0.36–0.70
300	976 ± 13	54.4 ± 0.5	0.50–0.74
320	1397 ± 19	45.8 ± 0.5	0.50–0.76

Interestingly, the range of variation of the *β*_KWW_ exponent is similar for all measurements. In fact, we found that the shape of the α-relaxation peak was basically the same for different (*T*, *P*) pairs having identical τ_α_ relaxation time value (isochronal condition). In other words, the distribution of relaxation times is found to depend only on the characteristic relaxation time, and not separately on *P* or *T*. This is shown in panels (c) and (d) of Fig. 2 for two distinct isochronal primary relaxation times, namely τ_α_ = 1.18 s and τ_α_ = 1.63 · 10^−4^ s. At these two values of τ_α_, the stretched exponent is found to be equal to 0.60 ± 0.02 and 0.64 ± 0.01, respectively, for all corresponding (*P*,*T*) pairs. The isochronal superposition of the loss peak for different values of τ_α_ is quite remarkable, and it is not at all trivial, because *β*_KWW_ decreases significantly upon approaching the glass transition. These results show that a generalized isochronal temperature-pressure superposition principle applies to TDZ, as typically occurs in non hydrogen-bonded systems^34^.

From the fits of the isobaric data it is possible to extract the fragility of the TDZ glass former, which is a measure of the degree of deviation from the simply-activated Arrhenius behaviour. The fragility is defined as^33, 35^:

5 $$m={\frac{{\rm{d}}(\mathrm{Log}{\tau }_{\alpha })}{{\rm{d}}({T}_{{\rm{g}}}/T)}|}_{T={T}_{{\rm{g}}}}$$

The fragility values are listed for all four isobaric measurements in Table 1, where it is seen that *m* is basically constant (actually, decreases slightly) with increasing pressure, with values ranging between 65 and 77. Therefore, TDZ can be classified as a fragile glass former according to Angell’s classification.

The roughly constant value of *m* also at elevated pressures indicates that the hydrogen bond scheme remains almost unchanged at high pressure, with at most a slight increase in the hydrogen-bond interactions. This finding is opposite to the usual behaviour observed *e*.*g*. for water^36^ and some monohydroxy alcohols^37, 38^, where the number of hydrogen bonds is lower at higher pressure.

**Figure 3:**
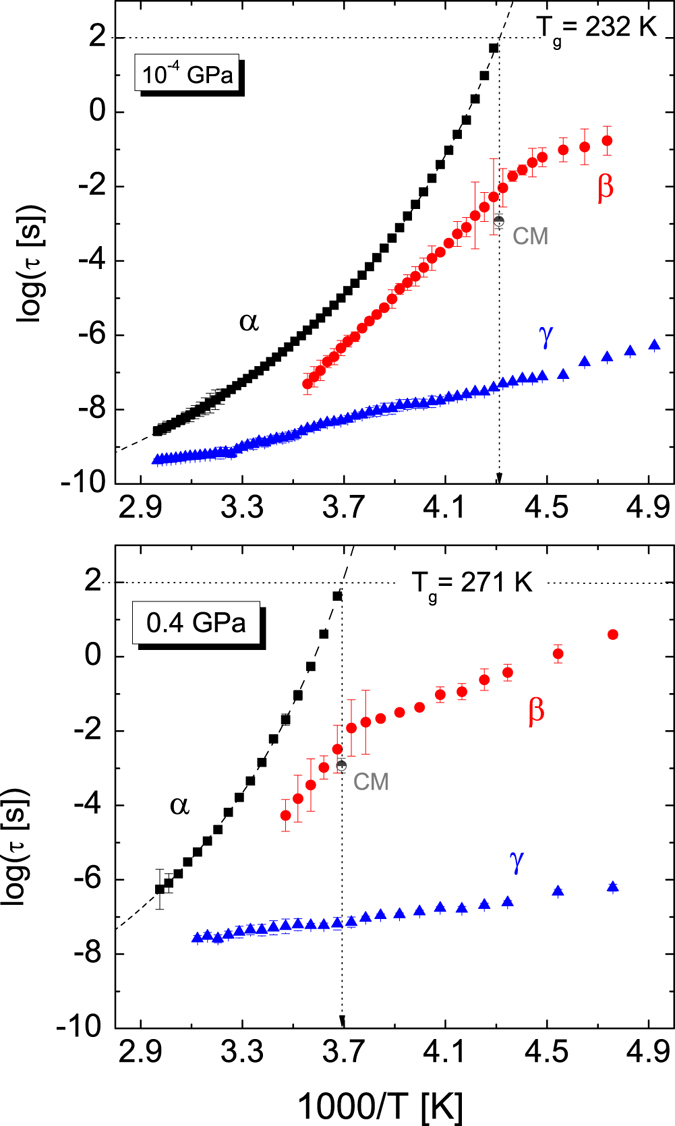
Arrhenius plot of the relaxation times for all three dynamic processes observed in TDZ in isobaric measurements, at ambient pressure (upper panel) and at 0.4 GPa (lower panel). Error bars are smaller than markers except where they are indicated. The grey half-filled circle with error bar corresponds to the precursor relaxation time near *T*_g_, calculated according to the Coupling Model.

**Figure 4:**
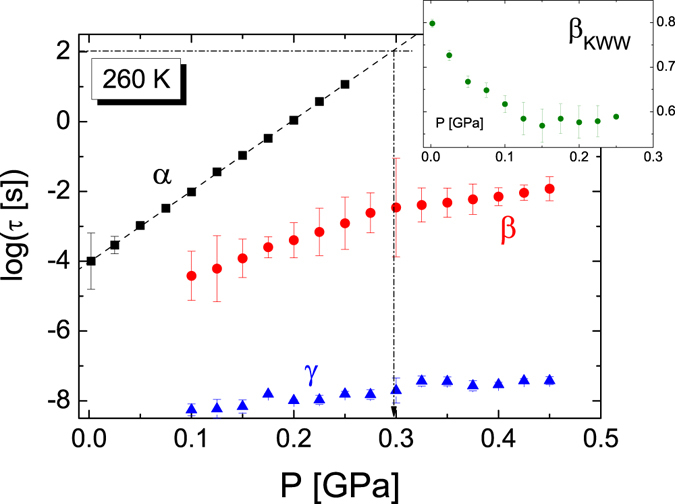
Relaxation times of the three different processes as function of pressure, for the isothermal measurements at 260 K. The vertical dashed line marks the value of *P*_g_ at this temperature. Insets: *β*_KWW_ stretched exponent as derived from the fit of the α relaxation peak, as function of pressure. Error bars are smaller than markers except where they are explicity drawn.

We now analyze the secondary relaxations and discuss their microscopic origin. The relaxation times of all three losses (α, *β*, γ), as obtained from the fits, are plotted as a function of the reciprocal of temperature (Arrhenius plot) at fixed pressure (ambient pressure and 0.4 GPa) in Fig. 3, and as a function of pressure in Fig. 4, at the fixed temperature of 260 K.

**Figure 5:**
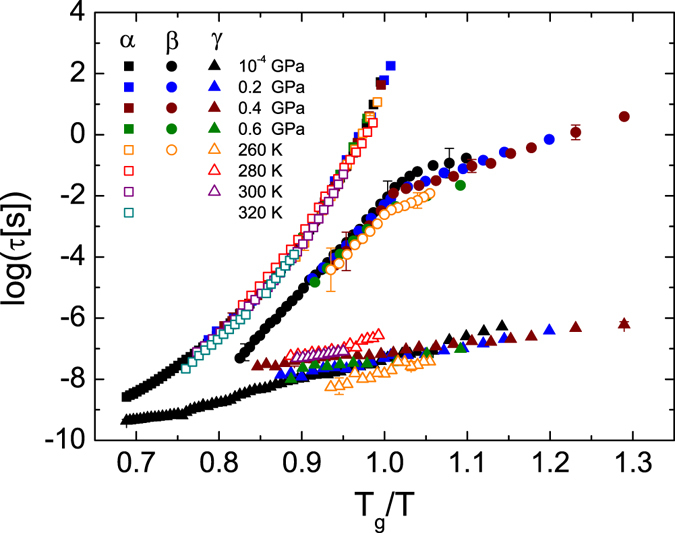
Master plot of the relaxation times for all three relaxation processes in TDZ, as a function of the reciprocal temperature normalized to the glass transition temperature (*T*_g_). Representative error bars are indicated. See the text for details.

The *β* relaxation has all the characteristic features of a Johari-Goldstein (JG) process^39^. A strong hint to the JG character of the *β* process is the presence of a characteristic cross-over in the slope parameter of the Arrhenius plot of *τ*_*β*_ at the glass transition temperature^40, 41^. Such cross-over is visible as a kink in both Figs 3 and 4, and it was observed in all experiments. Further proof of the JG nature of the *β* relaxation is the fact that its relaxation time scales with a function correlated to the α process. Figure 5 shows the superposition of the Angell plots for all *T*- and *P*-dependent measurements. In the figure, the relaxation times of the three dynamic processes are displayed as function of the reciprocal temperature normalized to the glass transition temperature *T*_g_(*P*) at the appropriate pressure value. To make such plot, for isobaric measurements the horizontal scale of each Arrhenius plot was simply rescaled by the corresponding *T*_g_. For isothermal measurements, we took each point in Fig. 2(b) and plotted it not as a function of *P* but rather as a function of the corresponding temperature, normalized to the value of *T*_g_ for the given pressure. The latter glass transition temperature was obtained from the Andersson and Andersson fit curve (Equation 3) to the *T*_g_ *vs P* curve shown in the inset to Fig. 2(a).

It may be observed from Fig. 5 that not only are all α relaxation curves superposed onto one another, as expected since the fragility is almost constant, but so are also all *β* relaxation curves. This means that the separation in frequency between the α and *β* process is constant for all (*P*, *T*) pairs, that is, that the two processes have actually the same origin. This is definite proof that the *β* process is the JG relaxation associated to the α process^42^. The similar slope of the Arrhenius plot of the *β* relaxation times below the glass transition in Fig. 5 suggests that the activation energy of this process in the glass state scales as *T*_g_(*P*), as observed in van der Waals systems^40–42^.

We have also checked whether the Coupling Model (CM)^43, 44^ holds for the *β*_JG_ process. According to such model, the *β*_JG_ process has nearly the same timescale of the primitive (precursor) motion initiating the cooperative α relaxation. It is predicted that the relaxation times of both relaxations are related as ${\tau }_{{\rm{CM}}}={({t}_{c})}^{1-{{\rm{\beta }}}_{{\rm{KWW}}}}{({\tau }_{\alpha })}^{{{\rm{\beta }}}_{{\rm{KWW}}}}$, where *τ*_CM_ is the predicted relaxation time according to the CM, *t*_c_ is a cross-over time typically equal to 2 · 10^−12^ s for organic glass formers^44, 45^, and *β*_KWW_ is the stretched exponent of the α relaxation. The value of *τ*_CM_ calculated for the experimental temperature closest to *T*_g_ is shown as a grey symbol with associated error bar in both panels of Fig. 3, along with the experimental relaxation times. A rough agreement is found between this value and the experimental *τ*_*β*_ value near *T*_g_.

As to the γ relaxation, it is found to be always the fastest process whatever temperature and pressure conditions. It is observed in Fig. 5 that the dependence of *τ*_γ_ upon the thermodynamic variables is not as pronounced as for the α and *β*_JG_ processes, and moreover that the γ relaxation does not obey a scaling law with the normalized temperature. This shows that the origin is independent of the α and *β*_JG_ processes^46^. Having assigned the *β* relaxation to a JG relaxation, the γ relaxation must correspond to an intramolecular dynamic process of the (non-rigid) TDZ molecules. Since such intramolecular dynamics has to be dipolar to be observed by dielectric spectroscopy, we assign the γ process to a relaxation of the propanol chain, which is likely involved in the formation of inter-molecular hydrogen bonds as in the crystal phase of TDZ^18^.

Having provided an assignment of all dynamical features of TDZ, we now show that the (*P*,*T*)-dependences of the α and *β* relaxation times fulfil the so-called thermodynamic scaling as a function of a single density-dependent parameter, namely the factor *Tv* ^γ^ (see Eq. 1), where *v* is the specific volume, with the same value of the exponent γ for both relaxations. To obtain such thermodynamic scaling, it is necessary to find the equation of state *v*(*P*,*T*) for TDZ, in order to calculate the volume at every pressure and temperature. The equation of state was obtained by fitting the curves of *v* vs *P* at four different temperatures (data obtained in PVT measurements, see the Experimental section) by means of the Tait equation^47^:

6 $$v(P,T)={v}_{0}[1+{\alpha }_{v}(T-{T}_{0})][1-kLn(1+\frac{P}{{b}_{1}exp(-{b}_{3}T)})]$$

**Figure 6:**
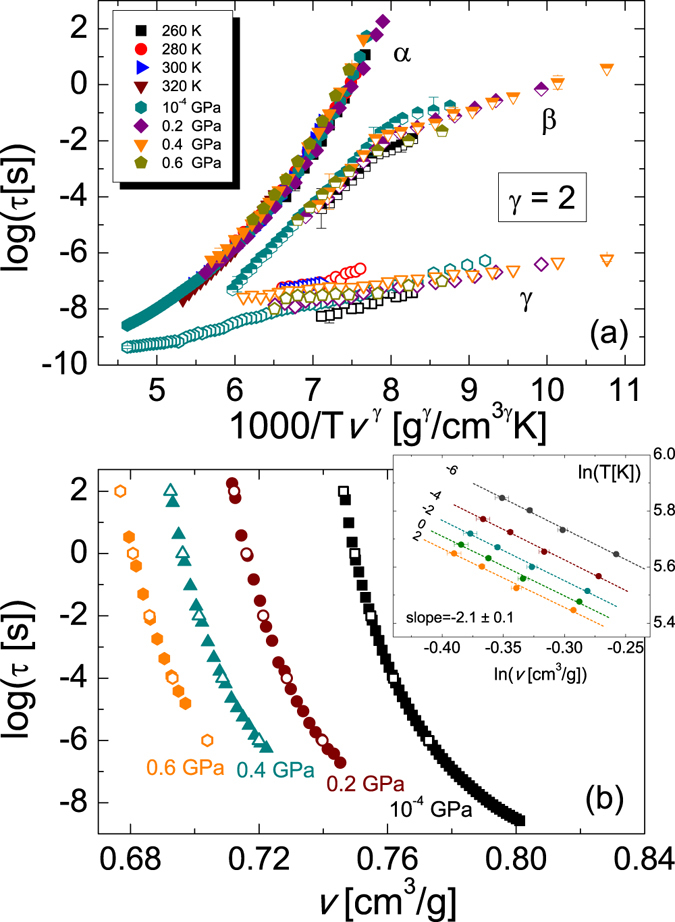
(**a**) Thermodynamic scaling plot of the relaxation times of all three dynamic processes, as function of the quantity 1000/*Tv* ^γ^ with γ = 2 (*v* is the specific volume calculated for each (*P*, *T*) pair by means of the experimental equation of state, Eq. 6). Representative error bars are indicated. (**b**) Plot of Log(*τ*_α_) as function of *v* for all four isobaric measurements. Filled markers correspond to the experimental data of Fig. 2(a). Open markers represent, for each isobar, the calculated value of *v* at selected relaxation times (namely *τ*_α_ = 10^−6^, 10^−4^, 10^−2^, 10^0^ and 10^2^ s). Inset: plot of ln(*T*) *vs* ln(*v*) for all four isobaric measurements (markers), for the five different isochronal conditions corresponding to the open markers in the main panel, and corresponding linear fit (dashed line).

The results of the *Tv* ^γ^ scaling of the relaxation time data are shown in Fig. 6(a). For completeness, we extended the scaling also to the data below the glass transition, although this extrapolation should be taken with caution, as the equation of state Eq. 6 is not expected to be valid in the glass phase.

A common scaling of all primary relaxation times in the supercooled liquid state (above the glass transition) is obtained with a thermodynamic exponent *γ* = 2.0 ± 0.1. It can be seen from the figure that the *β* relaxation scales with the same factor used for the α relaxation, corroborating once more the JG origin of this secondary relaxation. On the other hand, the relaxation times of the fastest process are not superposed onto one another, as expected considering the intramolecular character of this relaxation.

The thermodynamic scaling plot of Fig. 6(a) not only confirms that the secondary *β* relaxation is a JG precursor of the primary relaxation, but also shows that there exists an isomorphic invariance of the relaxation times in TDZ. According to a recent theory, in simple systems in which the virial and potential energy fluctuations are correlated, such as dense van der Waals liquids^3, 21^ and some crystals^3, 22^, there exist lines in the thermodynamic phase diagrams along which structure and dynamics are isomorphic. Along these lines, known as isomorph states, the overall dynamics (characteristic relaxation time, width of the relaxation-time distribution, etc.) is invariant, that is, it can be rescaled on a single master curve when plotted against suitable reduced units. For relaxation times, the suitable scaling factor is given by a specific-volume-dependent energy barrier divided by temperature, *i*.*e*., the factor *Tv* ^γ^.

Isomorphs theory has been tested via molecular dynamics simulation in several systems, including crystalline ones, and by experiments in supercooled liquids and liquid crystals^19, 20^. The results displayed in Fig. 6(a) show that TDZ is characterized by a common isomorphic invariance – with the same scaling parameter – of both whole-molecule relaxations (α, *β*_JG_). These results are non-trivial, because TDZ is characterized by important hydrogen bonding, so that it is not a priori expected to follow the behaviour typical of van-der-Waals liquids. The obtained value of the thermodynamic exponent *γ* ≈ 2 is intermediate between the values typical of van der Waals systems, which range from 4 to 8, and the values smaller than unity reported for hydrogen network forming systems such as sorbitol and glycerol^4^.

The exponent *γ* of the thermodynamic scaling can also be estimated as the slope of the logarithmic plot of *T*_g_ *vs v*_g_^48^, where *v*_g_ is the specific volume at the glass transition, or in general from the slope of ln(*T*) vs ln(*v*) under isochronal conditions^38^. Figure 6(b) shows the semilogarithmic plot of *τ*_α_ for each of the four isobaric measurements (Fig. 2(a)), as function of the specific volume *v*. The latter was calculated for each experimental (*P*_isobar_, *T*) pair by means of the Tait Eq. 6 (*P*_isobar_ denotes the fixed pressure of one of the four isobaric data set). For each isobaric measurement, the temperature *T*_*τ*_(*P*_*isobar*_) at which the primary relaxation time has a given value *τ* can be determined by means of the VFT Eq. 2, with the parameters obtained from the fits to the data of Fig. 2(a). Similarly, the specific volume *v*_*τ*_(*P*_*isobar*_) at a given primary relaxation time *τ* along an isobar can be calculated inserting in Eq. 6 the pair of thermodynamic parameters (*P*_*isobar*_, *T*_*τ*_(*P*_*isobar*_)). The inset to Fig. 6(b) shows the logarithmic plot of the temperature *T*_*τ*_(*P*_*isobar*_) *versus* specific volume *v*_*τ*_(*P*_*isobar*_) for each isobar at five fixed isochronal times of the primary relaxation, namely *τ* = 10^−6^, 10^−4^, 10^−2^, 10^0^ and 10^2^ s (the latter plot, for *τ* = 10^2^ s, is thus the logarithmic plot of *T*_g_ *vs v*_g_ at different pressures). A roughly linear dependence is observed for all isobars, with average slope *γ* = 2.1 ± 0.1. This value is consistent with the estimate of *γ* obtained from the master plot of Fig. 6(a).

Another independent way to estimate *γ* is to apply the following relation^13^ (Eq. 7) connecting the values of several static thermodynamic quantities measured on crossing the glass transition at atmospheric pressure:

7 $$\gamma ={[\frac{{\rm{\Delta }}{C}_{P}{\kappa }_{T}}{{\rm{\Delta }}{\alpha }_{P}{V}_{g}}-{\alpha }_{P}{T}_{{\rm{g}}}]}^{-1}$$

For ternidazole *α*_*P*_ = 6.99 · 10^−4^ K^−1^, *Δα*_*P*_ = 4.33 · 10^−4^ K^−1^, *ΔC*_*P*_ = 101.6 J mol^−1^ K^−1^, *κ*_*T*_ = 3.90 · 10^−4^ MPa^−1^, and *V*_*g*_ = 138 cm^3^ mol^−1^, which results in γ = 2.0 ± 0.2, in good agreement with the scaling exponent.

It is worth emphasizing that such value for *γ* is the lowest ever reported for a molecular glass-former obeying, over a wide *T*-*P* interval, (*i*) thermodynamic scaling, (*ii*) isochronal loss peak invariance, and (*iii*) co-invariance of α and *β*_JG_ relaxation times. Although polyols (glycerol and sorbitol) and glycols show lower γ values than TDZ^4, 10^, the thermodynamic scaling applies in such cases only over a limited range, and upon increasing the pressure the fragility index increases strongly^49^ and the isochronal loss peak broadens and the α and *β*_JG_ relaxation times become more separated^50^. The reported origin of the low value of *γ* in polyols is the progressive weakening and disruption of the highly directional hydrogen bonds upon increasing *T* or *P*, leading to different molecular arrangement and interactions and to a strong dependence of the dynamics on thermal fluctuations, with the density playing only a minor role. In case of TDZ, since the isomorphism scenario applies over the whole range of temperature (30%) and density (15%) variations applied, the intermolecular interactions are apparently not modified for different thermodynamic conditions. We suggest that the low value of γ in TDZ could be due to the strong dipole moment associated to the nitroimidazole ring, as dipole-dipole interactions enhance^51^ the attractive part of the intermolecular pair potential thus increasing the sensitivity to thermal fluctuations and consequently reducing the value of the thermodynamic scaling exponent.

We finally point out that the thermodynamic scaling of Fig. 6(a) bears a striking resemblance with the *T*/*T*_g_(*P*) scaling (generalized Angell plot) of Fig. 5. This resemblance is likely another consequence of the self-isomorphic behaviour. Whichever the origin of the similarity, it is worth emphasizing that the scaling of Fig. 6(a) has a more profound meaning than that of Fig. 5: while the Angell plot shows that the (*P*,*T*)-dependence of the dynamics in a given system is determined by how far in the (*P*,*T*) plane the system is from the glass transition, the thermodynamic scaling shows that such distance is a function of a single scaling parameter that depends on the specific volume, *i*.*e*., on the intermolecular distance. The data for the γ relaxation indicate that such behaviour is not instead shared by the intramolecular motions, which have lower activation volume and are therefore not influenced by the change in intermolecular distance brought about by density variations. The γ relaxation time is likely determined by bond rotational energy barriers which do not depend strongly on density.

To summarize, we have employed temperature- and pressure-dependent dielectric spectroscopy to probe the dipolar dynamics of amorphous ternidazole, both above and below the glass transition temperature *T*_g_. The equation of state, as determined from pressure-temperature-volume measurements, can be described by means of the Tait equation. Three molecular dynamic processes are identified, two of which are whole molecule dynamics. The whole-molecule dynamic processes are the α relaxation, whose characteristic time is equal to 100 s at the glass transition, and the related Johari-Goldstein *β*_JG_ relaxation. The third dynamic process is instead associated with the motion of the propanol chain of the molecule. We observe several features that reveal the existence of dynamic isomorphism within the temperature and pressure ranges experimentally available. On one hand, the lineshapes of dielectric loss spectra characterized by the same α relaxation time are virtually identical to one another. On the other, both α and *β*_JG_ relaxation times are found to follow a common scaling behaviour when plotted against the normalized temperature *T*/*T*_g_ and also when they are displayed against the thermodynamic quantity *Tv* ^*γ*^ with *γ* = 2. The validity of the latter thermodynamic scaling is particularly intriguing, as it is usually reported for van-der-Waals liquids or crystals and not for hydrogen-bonded systems such as ternidazole. The relatively low value of γ, which has been checked using three independent methods, indicates that thermal fluctuations have a stronger impact on the dynamics than density changes.

## Experimental Section

Medicinal grade ternidazole (TDZ) was provided by Bouchara-Recordati, Levallois Perret (France) and used as received. The material is the same as that used in ref. 18. Pressure-volume-temperature measurements^52^ on TDZ were carried out at 368.2, 356.2, 346.2, 325.2 and 303.2 K, in a pressure range between 0.1 and 0.3 GPa.

Temperature-dependent broadband dielectric spectroscopy measurements were performed at ambient and high pressure with the sample sandwiched inside a parallel-plate capacitor, into which TDZ was inserted by heating it to reach the liquid (molten) state. The spectra at ambient pressure were taken between 10^−2^ and 10^9^ Hz, in the temperature range between 203 K and 337 K every 2 K. Pressure-dependent dielectric measurements were carried out in the frequency range between 10^−2^ and 10^7^ Hz. For the measurements between 10^−2^ and 10^7^ Hz a Novocontrol Alpha Analyzer was employed, while for measurements between 10^7^ and 10^9^ Hz a HP4291 impedance analyzer was employed, in reflectometry geometry with the sample capacitor mounted at the end of a coaxial cable. For the measurements at high pressure, in order to prevent a possible contamination with the pressurizing fluid (thermal oil from Huber) the capacitor was covered by a teflon membrane and latex wrapping. The insulated capacitor was then placed in a high-pressure chamber (Unipress) made of a Cu-Be alloy, which was filled with the thermal oil and connected to a manual pump that allowed applying hydrostatic pressure between ambient pressure and 0.6 GPa, as measured by means of a pressure transducer with an accuracy of ±0.5%. The temperature was controlled by thermal baths (Lauda Proline RP 1290 and Huber Unistat) with a liquid - flow circuit connected to the high-pressure setup. Isothermal pressure scans were taken at 260, 280, 300 and 320 K, and isobaric measurements at normal pressure and 0.2, 0.4 and 0.6 GPa were performed in the temperature range from 210 K to 336 K.
